# Zoonotic Genotypes of *Enterocytozoon bieneusi* in Wild Living Invasive and Native Carnivores in Poland

**DOI:** 10.3390/pathogens10111478

**Published:** 2021-11-13

**Authors:** Agnieszka Perec-Matysiak, Kinga Leśniańska, Katarzyna Buńkowska-Gawlik, Dorota Merta, Marcin Popiołek, Joanna Hildebrand

**Affiliations:** 1 Department of Parasitology, Faculty of Biological Sciences, University of Wrocław, 51-148 Wrocław, Poland; kinga.lesnianska@gmail.com (K.L.); katarzyna.bunkowska-gawlik@uwr.edu.pl (K.B.-G.); marcin.popiolek@uwr.edu.pl (M.P.); joanna.hildebrand@uwr.edu.pl (J.H.); 2 Department of Ecology and Environmental Protection, Institute of Biology, Pedagogical University of Kraków, 30-084 Kraków, Poland; dorota.merta@up.krakow.pl

**Keywords:** *Enterocytozoon bieneusi*, zoonotic genotypes, raccoon dog, raccoon, carnivores

## Abstract

Wild carnivores, both introduced and native species, are able to adapt well to peri-urban environments, facilitating cross-species pathogen transmission with domestic animals, and potentially humans. The role of wild living reservoir hosts cannot be ignored because of their known carriage of *E. bieneusi* zoonotic genotypes. In the past decades, populations of wild living carnivores, i.e., native, such as red foxes, and invasive, such as raccoon dogs and raccoons, have increased and adapted to synanthropic environments across Europe, including Poland. The knowledge concerning *E. bieneusi* genotype identification and distribution in wild carnivores is limited worldwide. A total of 322 individual fecal samples from six carnivore species, i.e., raccoon, raccoon dog, red fox, European badger, pine and beech martens, were collected and then analysed for the presence of *E. bieneusi* using the nested PCR method. Overall prevalence of the pathogen was estimated to be as high as 27.3%. The infection rates for *E. bieneusi* varied between the carnivore species, from 13.7% in beech martens to 40.4% in raccoon dogs. Based on sequence analysis of the ITS region of the rRNA gene marker, we detected five known genotypes of *E. bieneusi* in examined animals. In the invasive species, *E. bieneusi* NCF2 and D genotypes have been identified, whereas in the native ones, *E. bieneusi* NCF2, D, C, EbCar2 and Type IV genotypes were identified. All *E. bieneusi* genotypes recorded in this survey clustered in Group 1, showing their zoonotic potential. Our results provide the first description of the occurrence and genotypes of the microsporidian *E. bieneusi* in wild living population of raccoon dogs in Europe. Our findings are important for the study of pathogen epidemiology and emphasize the fact that the invasive and the native wild living carnivores, both widely distributed, should be considered more seriously as significant sources of zoonotic pathogens hazardous to domestic and farmed animals and humans.

## 1. Introduction

*Enterocytozoon bieneusi* is a microsporidian species found ubiquitously in both invertebrate and vertebrate hosts in many different environments including domestic and wild animals, and humans [1]. Both immunocompetent and immunocompromised individuals are at risk of *E. bieneusi* infection. Infection by *E*. *bieneusi* may have no symptoms or may cause persistent diarrhoea, vomiting and a wasting syndrome, particularly in immunocompromised patients. To date, microsporidian infections have been observed in a wide range of human populations, including autoimmune diseases, end-stage renal failure, human immunodeficiency virus (HIV)-positive individuals, leukemia patients and travelers [2,3]. Ubiquitous microsporidia occurrence in animal hosts and water sources affects the potential risk of human microsporidiosis [3]. At present, over 500 *E. bieneusi* genotypes have been identified in humans, animals and environmental samples [4]. Phylogenetic analysis of *E. bieneusi* internal transcribed spacer (ITS) sequences revealed the presence of eleven groups of genotypes, which display varying degrees of host specificity and zoonotic potential. Groups 1 and 2 contain genotypes most frequently found not only in humans, but also in domestic and wild animals worldwide. *E. bieneusi* genotypes in Groups 3 to 11 are genetically divergent and mostly restricted to a particular host range and thus represent a minor or unknown public health threat [3]. 

A continuous anthropogenic alteration of natural environments, comprising a progressive urbanisation as well as the use of natural habitats for agriculture, forestry or recreational uses, has been observed in Europe during the last decades. Furthermore, the fragmentation and the destruction of natural habitats provoke an increasing contact of wildlife with domestic animals, as well as humans, entailing the risk of pathogen spillovers from sylvatic to domestic or synanthropic cycles [5,6]. The occurrence of a variety of zoonotic pathogens in wild animals raises a number of issues with major implications for domestic animals and human welfare. Animals living in the wild share many infectious agents with humans and domestic animals, and the origins and direction of the pathogen spillover often remain unclear. Wild carnivores, both introduced and native species, are able to adapt well to peri-urban environments, facilitating cross-species pathogen transmission with domestic animals, and potentially humans. The role of wild living reservoir hosts, cannot be ignored because of their known carriage of *E. bieneusi* zoonotic genotypes [3]. In the past decades, populations of wild living carnivores, i.e., red foxes (*Vulpes vulpes*) and raccoon dogs (*Nyctereutes procyonoides*), as well as raccoons (*Procyon lotor*), have increased and adapted to synanthropic environments across Europe, including Poland [7,8]. The expansion success of alien species such as raccoons and raccoon dogs is attributed to their ability to adapt to several environments, their omnivorous feeding habits, high reproductive potential and lack of natural enemies. Their geographical ranges with a suitable climate for raccoons and raccoon dogs widely overlap in Europe. The raccoon uses human settlements, where it can cause damage to buildings and houses by looking for resting sites. This mammal has been observed readily utilizing anthropogenic resources, such as refuse for food and human structures for dens [9]. One of the most important impacts these species have in their non-native range in Europe is their potential as health hazards for humans and animal livestock [10,11].

The raccoon is a North American carnivore, which was introduced in the 20th century to Japan and Europe, including Poland [12]. The presence of raccoons near human settlements may pose a threat to human health. As research conducted in the US proved, this animal is host to numerous species of parasites and pathogens [10]. Despite the fact that the raccoon is an alien and invasive species, both wild living and potentially synanthropic, which has been present in Europe for over 80 years, the knowledge concerning its parasitofauna is still insufficient, especially concerning microparasites. Several studies concerning the European population of raccoons have so far revealed the presence of zoonotic genotypes of *Cryptosporidium* sp. and *E. bieneusi* [13,14], helminth species and vector-borne pathogens [15,16]. The raccoon dog, an invasive alien species introduced from the Far East to the European areas of the former Soviet Union in the mid-twentieth century [17], is rapidly urbanizing, successfully adapted to most habitats and able to quickly achieve a high population density. Not only does the raccoon dog negatively influence the biodiversity of newly colonized areas, more importantly, it may serve as a reservoir for zoonotic agents, which affect native co-occurring species such as the red fox, badger and marten [7,18]. The red fox is the most common wild canid in Europe with a high population density and wide geographical distribution, including highly urbanized areas [19,20].

The knowledge concerning the raccoon as reservoir hosts of *E. bieneusi* is limited and concerns native territories of Central and North America [21,22]. Data regarding the prevalence and genetic diversity of *E*. *bieneusi* in European populations of invasive raccoons are only limited to our preliminary study carried out previously in Poland and Germany [13]. The majority of studies investigating the occurrence of zoonotic and potentially zoonotic genotypes of *E*. *bieneusi* in raccoon dogs and foxes have focused on animals farmed for their fur in China and Korea [23,24,25,26,27,28,29] and one study concerning wild living foxes has been carried out in the US [21]. To the best of our knowledge, no data are available concerning the occurrence of this pathogen in invasive raccoon dogs in Europe, despite their wide distribution and population density. In Europe, there have been two studies concerning *E. bieneusi* occurrence in wild living mesocarnivores, such as the red fox, badger and marten carried out in Spain [30,31]. Therefore, the objectives of this study were (1) to understand the distribution of *E. bieneusi* genotypes in wild invasive and native carnivores with overlapping ranges; (2) to examine possibility of cross-species transmission of *E. bieneusi* genotypes between invasive and native species; and 3) to assess the zoonotic potential of *E. bieneusi* in examined animals. 

## 2. Results

**Figure 1:**
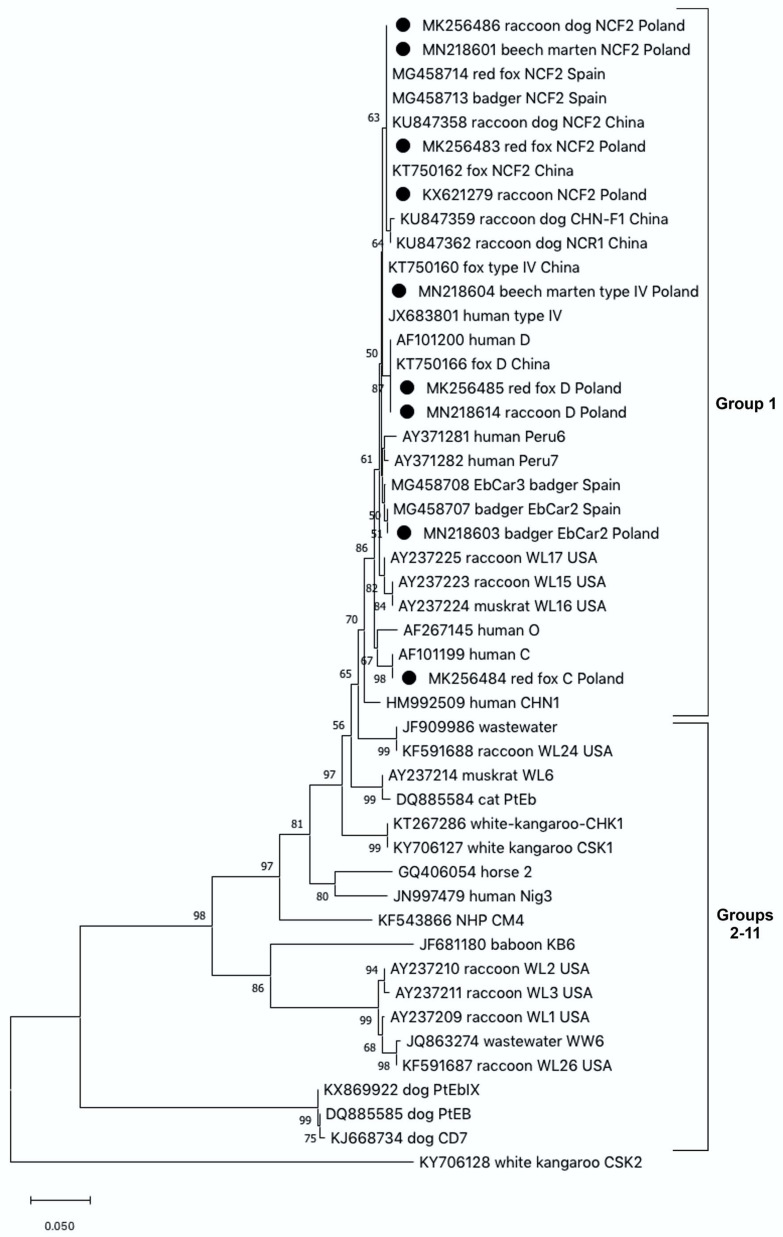
The phylogenetic relationships among *Enterocytozoon bieneusi* genotypes identified in wild carnivores in this study and others as inferred by a neighbor-joining analysis of the ITS region of the rRNA gene sequences implemented in MegaX. Bootstrapping was performed using 1000 replicates; values below 50% are not shown. Sequences from this study are marked with solid circles. In the cases of carnivore isolates, the branch label includes accession number, followed by animal host, genotype name and country of origin.

The fecal samples obtained from examined mesoranivores were analysed for the presence of *E. bieneusi* using the nested PCR method (Table 1). Overall prevalence of the pathogen was estimated as high as 27.3%. The infection rates for *E. bieneusi* varied between the carnivore species, from 13.7% in beech martens, 16.7% in pine martens, 23.0% in raccoons and 30% in red foxes to 40.4% in raccoon dogs. Based on sequence analysis of the ITS region of the rRNA gene, we detected five known genotypes of *E. bieneusi*. Sequence analyses revealed that the majority of *E. bieneusi* positive isolates were of the NCF2 genotype, identified in all examined species, with the exception of a European badger. Phylogenetic analysis showed that the identified NCF2 genotype was identical to those previously reported in the red fox (Acc. No. MG458714) and the European badger (Acc. No. MG458713), in Spain, as well as in the fox, (Acc. No. KT750162) and raccoon dog (Acc. No. KU847358), in China. Zoonotic *E. bieneusi* genotype D was identified in the samples obtained from the raccoon and red fox, whereas *E. bieneusi* genotype C was identified only in one sample derived from the red fox. These isolates were identical to those previously identified in humans (Acc. No. AF101200 and Acc. No. AF10199, respectively). The nucleotide sequences obtained from the European badger showed 100% homology with *E. bieneusi* genotype EbCar2 (Acc. No. MG458707), and 99.7% with genotype EbCar3, (Acc. No. MG458710) which were obtained from the European badgers from Spain. In martens, *E. bieneusi* NCF2 and type IV genotypes were identified. The sequence of the *E. bieneusi* type IV genotype was identical to the one previously published in the GenBank database, identified, e.g., in a human from Nigeria (Acc. No. JX683801), and in a fox from China (Acc. No. KT750160). All *E. bieneusi* genotypes recorded in this survey were clustered in Group 1 showing their zoonotic potential (Figure 1).

## 3. Discussion

Molecular epidemiological data demonstrating the occurrence of *Enterocytozoon bieneusi* in carnivorous species derive mostly from studies carried out on farmed animals. Relatively little is known about the distribution of zoonotic *E. bieneusi* genotypes in European wild carnivores, including the invasive species. The raccoon and raccoon dog, regarded as the most successful invasive alien carnivores, have established flourishing self-sustaining populations in Europe, and their further expansion is still in progress [7]. The red fox is the most common wild species among canids in Europe [32] and often co-occurs with mustelids such as martens and badgers. These carnivore species, native and invasive, contaminate their foraging places with their respective infectious agents [33]. Since their home ranges overlap, opportunities occur for these pathogens to switch and adapt to new host species. This makes it possible to note the cross-species transmission of microparasites occurring in areas cohabited by these animals.

In this study, based on sequence analysis of the ITS region of the rRNA gene marker, we detected five known genotypes of *E. bieneusi* in examined species of carnivores. In the invasive species, only *E. bieneusi* NCF2 and D genotypes were identified. On the other hand, a greater number of genotypes such as *E. bieneusi* genotype NCF2, D, C, EbCar2 and Type IV were detected in the native carnivores. Our survey provides the first data documenting the presence of *E. bieneusi* in wild living and invasive raccoon dogs in Europe. We found out that 40.2% of raccoon dogs examined were infected with this microsporidium and only the *E. bieneusi* NCF2 genotype (syn. WildBoar3, NCF3 and NCF4) was recorded in the positive samples. The *E. bieneusi* NCF2 genotype appears to be widespread in European wildlife, having also been found in other mesocarnivore species such as the European badger in Spain and in wild boars in the Czech Republic and Poland [31,34]. During surveys carried out in China in farmed raccoon dogs, a lower prevalence of 2.6–22% has been reported, and a great number of *E. bieneusi* genotypes both known and novel, including the NCF2 genotype, were identified [23,24,26,28,29]. A rather high prevalence of 35.4%, comparable to those obtained in this study, was recorded in raccoon dogs obtained from wildlife centres in Korea [27]. It is probably the social behaviour of the raccoon dogs that facilitates contact among families and other groups of infected animals co-occurring in the same habitat, which may be responsible for the high infection rate detected in the present study. Based on the results of our study, we observed that during the establishment of new populations of raccoon dogs in Europe, this canid lost many of the *E. bieneusi* genotypes, recorded earlier in their native areas. In North America, in raccoons, the variety of human pathogenic genotypes such as Peru 11, EbpC, WL15 and D as well as genotypes adapted to raccoons such as WL1-3, WL13, WL15-17, WL24, WL26 and WW6 were identified [21,22]. In addition, the study undertaken in the US showed a relatively high (82%) infection rate of *E. bieneusi* in raccoons [22] compared with those obtained in the introduced area in Poland, 23% (this study) and 4.1% [13]. Thus, the raccoon is another example of an invasive species, which has lost most of its *E. bieneusi* genotypes but on the other hand newly accumulated the *E. bieneusi* NCF2 genotype, widely distributed among European wildlife [26]. This agrees with a study by Torchin and Mitchell [35], which shows that a species introduced into a novel environment not only loses its own parasites but can also accumulate parasites already present in the newly colonized environment. Additionally, we recorded the presence of genotypes D in several isolates derived from raccoons. *E. bieneusi* genotype D is distributed across a wide range of hosts, including humans, and is considered a zoonotic genotype with public health significance [36]. 

In Europe, the only studies so far demonstrating *E. bieneusi* presence in wild carnivores were limited to foxes, martens and badgers in Spain [30,31], where it was found in 9.2% to 23.2% of the animals examined. Alarmingly, in this study we found the prevalence of this microsporidium in wild red foxes from Poland to be as high as 30%. A similarly high prevalence of *E. bieneusi* (27.7%) has been recorded in farmed red foxes in China, and has been attributed to the greater abundance of spores due to the increased density of foxes on farms [23]. In Poland, as well as in other European countries, increased fox populations have been reported, which may explain the high infection rate of the pathogen [20]. The animal-specific *E. bieneusi* genotypes NCF2, EbCar4 and S9, which clustered in zoonotic Group 1, have been identified in foxes in Spain [26]. In our study, we also found a potentially zoonotic genotype, NCF2, in a majority of the isolates and genotype D in two isolates obtained from foxes. In addition, the *E. bieneusi* C genotype was recorded in one isolate derived from a red fox. Until now this genotype has been identified only in human samples from Switzerland, Germany, the Netherlands and France [2], thus our report is the first to demonstrate the occurrence of this pathogen in a wild animal. 

The prevalence of *E. bieneusi* in the badgers obtained in this study was similar to that described in the Spanish study and reached 26.7%. The Spanish badgers showed the genetic diversity of identified *E. bieneusi* genotypes and NCF2 genotype was the most common in badgers in that survey. In our study, the *E. bieneusi* EbCar2 genotype was the only one identified in badgers. The genotype is one of four (EbCar1–EbCar4) genotypes newly described in wild mesocarnivores from Spain [31]. In the martens examined, the prevalence of *E. bieneusi*, was similar to that obtained in the Spanish survey and reached 16.7% for pine martens and 13.7% for beech martens. *E. bieneusi* NCF2 and type IV genotypes were identified, whereas *E. bieneusi* EbCar1 was the only identified genotype in one beech marten in Spain [31]. The zoonotic genotype *E. bieneusi* type IV has been described in pine martens in our own research and in other species of wild mammals, which suggests that it may be widely distributed in the environment; therefore, it shows a high potential for cross-species transmission between humans and wildlife [2,29]. 

In conclusion, we have provided an analysis of the prevalence of *E. bieneusi* in the wild populations of carnivores in Poland, Europe, and uncovered a very high incidence of the occurrence of zoonotic *E. bieneusi* genotypes in all of the species examined. Our results provide the first description of the occurrence and genotypes of the microsporidian *E. bieneusi* in wild living population of raccoon dogs in Europe. Moreover, we identified the red fox as a carrier of *E. bieneusi* genotypes D and C, which are pathogenic for humans. It is of note that the latter has never before been isolated from an animal sample. These findings are important for the study of pathogen epidemiology and emphasize the fact that the invasive and the native wild living carnivores, both widely distributed, should be considered more seriously as significant sources of zoonotic pathogens hazardous to domestic and farmed animals and humans.

## 4. Materials and Methods

### 4.1. Study Areas and Specimen Collection 

A total of 322 individual fecal samples from six carnivore species, raccoon (n = 65), raccoon dog (n = 86)*,* red fox (n = 50), European badger (45), pine martens (24) and beech martens (51), were collected during the period 2017–2019. All samples were obtained during necropsy from animals shot during a predator control operation or road-killed animals from Ruszów Forestry (51°24’00.1” N 15°10’12.2” E), which is located in the western part of the largest lowland forest complex in Europe, where, uniquely, the native and invasive carnivore species co-occur in the same habitat. All samples were kept at −20 °C until further analysis.

### 4.2. Molecular and Phylogenetic Analyses

DNA was isolated from all fecal samples using the GeneMATRIX Stool DNA Purification Kit (EURx, Gdańsk, Poland) according to the manufacturer’s protocols. The obtained isolates of DNA were stored at −20 °C until molecular analysis was carried out. PCR amplification was performed in a T100 Thermal Cycler (BioRad, Hercules, CA, USA) on a set of nested primers amplifying the ITS region of the rRNA gene of *E. bieneusi*, i.e., EBITS3 and EBITS4, and EBITS1 and EBITS2.4, with cycling parameters elaborated by Buckholt et al. [37]. All PCR amplifications were performed in 25 μL reaction volume, consisting of 5 μL template DNA, 12.5 μL of the standard and ready-to-use PCR mixture 2× PCR Mix Plus (A@A Biotechnology, Gdańsk, Poland), 0.25 μL of each primer (10 mM), 1.0 μL MgCl_2_ (25 mM), 1.0 μL BSA (50 mg/mL) and 5.0 μL of ddH_2_O for the first reaction. For the secondary PCR step, the PCR mixture was identical except that BSA was excluded and PCR product from the first run instead of template DNA was containing. For all PCR reactions, negative and positive controls were performed with sterile water and reference DNA isolated from *E. bieneusi* genotype D spores, respectively. Secondary PCR products were resolved by electrophoresis in a 1.0% agarose gel and stained with Simply Safe (EURx, Gdańsk, Poland). Chosen amplicons of the expected size were purified using Exo-BAP (EURx, Gdańsk, Poland) and stored at 4 °C until sequenced. Products were sequenced using an Applied Biosystems ABI PRISM 3100-Avant Sequencer (SEQme, Dobříš, the Czech Republic). The nucleotide sequences obtained were edited with DNA Baser Sequence Assembly software (Heracle BioSoft SRL, Mioveni, Romania) and aligned with reference sequences of *E. bieneusi* available in GenBank.

Phylogenetic analyses were performed using MEGAX software [38]. Trees were inferred by the neighbor-joining (NJ) method based on the Kimura 2-parameter distance model; bootstrapping was performed using 1000 replicates. Sequences obtained in this study were deposited in the GenBank database under the accession numbers MN218601-604, MN218614, and MK256483-MK256486.

## Figures and Tables

**Table 1:** Prevalence and distribution of *Enterocytozoon bieneusi* genotypes in raccoons, raccoon dogs, red foxes, badgers and martens obtained in this study and worldwide.

Carnivore Species	Collection Country	No. Examined/ No. Positive (%)	*E. bieneusi* Genotypes	References
Raccoon *Procyon lotor*	USA–native	55/15 (27.3)	D (1), WL1 (4), WL2 (2), WL3 (1), WL15 [syn. WL16] (4), EbpC [syn. WL17] (3)	Sulaiman et al., 2003 [21]
	USA–native	22/18 (81.8)	WL4 (8), WW6 (7), Peru11 (1), WL24 (1), WL26 (1)	Guo et al., 2014 [22]
	Germany–invasive (wild)	17/0	-	Leśniańska et al., 2016 [13]
	Poland–invasive (wild)	32/2 (6.3)	NCF2 (2)	Leśniańska et al., 2016 [13]
	Poland–invasive (wild)	65/15 (23)	D (4), NCF2 (11)	This study
Raccoon dog *Nyctereutes procyonoides*	China–native (farmed)	39/1 (2.6)	EbpA (1)	Zhang et al., 2021 [29]
	China–native (farmed)	305/68 (22.3)	D (9), CHN-DC1 (9), NCF2 (32), CHN-F1 (3), NCR2 (5), NCR1 (2)	Xu et al., 2016 [26]
	China–native (farmed)	49/2 (4.1)	D (1), CHN-R1 (1)	Zhao et al., 2015 [24]
	China–native (farmed)	162/17 (10.5)	CHN-DC1 (2), D (14), WildBoar3 (1)	Yang et al., 2015 [23]
	China–native (farmed)	356/23 (6.5)	CHG1 (1), D (8), Peru8 (3), Type IV (11)	Ma et al., 2020 [28]
	Korea–native (wildlife center)	48/17 (35.4)	Korea-WL1 (8), Korea-WL2 (6), Korea-WL3 (1), Korea-D (6)	Amer et al., 2019 [27]
	Poland–invasive (wild)	86/35 (40.2)	NCF2 (35)	This study
Red fox *Vulpes vulpes*	USA (wild)	67/9 (13.4)	D (2), WL11 (1), EbpC [syn. WL13] (3), WL15 (3)	Sulaiman et al., 2003 [21]
	Spain (wild)	7/1 (14.3)	D (1)	Galván-Díaz et al., 2014 [30]
	Spain (wild)	87/8 (9.2)	NCF2 (6), EbCar4 (1), S9 (1)	Santin et al., 2018 [31]
	China (farmed)	191/53 (27.7)	D (53)	Yang et al., 2015 [23]
	Poland (wild)	50/15 (30)	NCF2 (12), D (2), C (1)	This study
European badger *Meles meles*	Spain (wild)	69/16 (23.2)	PtEbIX (1), EbCar2 (5), NCF2 (4), S5 (5), EbCar3 (1),	Santin et al., 2018 [31]
	Poland (wild)	45/12 (26.7)	EbCar2 (12)	This study
Pine marten *Martes martes*	Poland (wild)	24/4 (16.7)	NCF2 (4)	This study
Beech marten *Martes foina*	Spain (wild)	9/1 (11.1)	EbCar 1 (1)	Santin et al., 2018 [31]
	Poland (wild)	51/7 (13.7)	NCF2 (5), Type IV (2)	This study
